# Governing the implementation of Emergency Obstetric Care: experiences of Rural District Health Managers, Tanzania

**DOI:** 10.1186/1472-6963-14-333

**Published:** 2014-08-03

**Authors:** Dickson Ally Mkoka, Angwara Kiwara, Isabel Goicolea, Anna-Karin Hurtig

**Affiliations:** 1 Department of Clinical Nursing, School of Nursing, Muhimbili University of Health and Allied Sciences, 901 85 Umeå, Sweden; 2 Department of Development Studies, School of Public Health and Social Sciences, Muhimbili University of Health and Allied Sciences, 901 85 Umeå, Sweden; 3 Department of Public Health and Clinical Medicine, Unit of Epidemiology and Global Health, Umeå University, 901 85 Umeå, Sweden

**Keywords:** Decentralization, Health reform, Health system governance, Emergency obstetric care, Council health management team, Tanzania

## Abstract

**Background:**

Many health policies developed internationally often become adopted at the national level and are implemented locally at the district level. A decentralized district health system led by a district health management team becomes responsible for implementing such policies. This study aimed at exploring the experiences of a district health management team in implementing Emergency Obstetric Care (EmOC) related policies and identifying emerging governance aspects.

**Methods:**

The study used a qualitative approach in which data was obtained from thirteen individual interviews and one focus group discussion (FGD). Interviews were conducted with members of the district health management team, district health service boards and NGO representatives. The FGD included key informants who were directly involved in the work of implementing EmOC services in the district. Documentary reviews and observation were done to supplement the data. All the materials were analysed using a qualitative content analysis approach.

**Results:**

Implementation of EmOC was considered to be a process accompanied by achievements and challenges. Achievements included increased institutional delivery, increased number of ambulances, training service providers in emergency obstetric care and building a new rural health centre that provides comprehensive emergency obstetric care. These achievements were associated with good leadership skills of the team together with partnerships that existed between different actors such as the Non-Governmental Organization (NGO), development partners, local politicians and Traditional Birth Attendants (TBAs). Most challenges faced during the implementation of EmOC were related to governance issues at different levels and included delays in disbursement of funds from the central government, shortages of health workers, unclear mechanisms for accountability, lack of incentives to motivate overburdened staffs and lack of guidelines for partnership development.

**Conclusion:**

The study revealed that implementing EmOC is a process accompanied by challenges that require an approach with multiple partners to address them and that, for effective partnership, the roles and responsibilities of each partner should be well stipulated in a clear working framework within the district health system. Partnerships strengthen health system governance and therefore ensure effective implementation of health policies at a local level.

## Background

Reducing maternal mortality and morbidity through improving maternal health services has been one of the global health challenges. A number of policies have been developed at international, regional and national levels describing strategies and interventions that focus on increasing access to quality and affordable maternal health services, especially in developing countries where over 99 percent of maternal deaths occurs [1,2]. Provision of antenatal care, training of traditional birth attendants on safe delivery and timely referral are examples of early strategies towards safe motherhood [3-5]. Today, access to facilities that offer emergency obstetric care (EmOC) with skilled birth attendants is the recommended strategy [5-9]. However, as is the case with other policies, implementation of maternal health policies is accomplished in the local setting and depends not only on technical input but also on mutual accountability and supportive interactions between communities, health facilities and the government [10].

### Implementing maternal health policies within decentralized health systems

Decentralization was a mechanism adopted in many developing countries to strengthen their health systems [11]. Health sector decentralization involved mainly deconcentration (transfer of responsibilities) and devolution (transfer of authority) [12-14] in a process aimed at increasing governance capacity to meet local health needs, increase the efficiency and quality of health services and court the participation of the community in making decision about their health [5,15-19]. Basically, decentralization and the broader health sector reforms were done with the purpose of improving health system performance [20-22]. Despite the influence of decentralization on service delivery, very little research has been done to explore its impact on delivery of quality health services. Existing literature indicates the benefits and detriments of decentralization on policy implementation. With regard to implementation of maternal health policies, decentralization was seen to be advantageous in integrating services at the local level, which is needed for safe motherhood programs [23]. However, it was also noted that decentralization resulted in poor quality of maternal health services, decreased coverage of immunization programmes, and weak supervision which led to decreased utilization of services [24,25]. Additionally, decentralization resulted in many players at the district level being responsible for maternal health policy implementation, making the system more complex and challenging to manage [26].

### A decentralized health system and implementation of Emergency Obstetric Care in Tanzania

Tanzania, like many developing countries, started the process of reforming various sectors in the early 1990s, with decentralization becoming the main component. Reforms in the health sector, local government and public service sector resulted in district health services coming under the responsibility of local government authorities. Local Government Authorities (LGAs) became responsible for planning, budgeting and managing health services, as well as other government services such as education, and the water supply [27]. In the health sector, decentralization resulted in the formation of council health management teams (CHMT) as leading organs of the district health system. The CHMT took over the operational functions of the Ministry of Health (MoH) while taking into account local district health priorities, needs and available resources [27,28]. This demands the CHMT to develop a strong and effective governance capacity [26].

Given the high maternal mortality rate in Tanzania and in response to its commitment towards improving maternal health, the Ministry of Health developed a number of policy documents and guidelines addressing delivery of quality emergency obstetric care. Most of these policies were developed in response to international and regional policies and agreements targeting the reduction of maternal deaths, such as the Delhi Declaration 2005 and Millennium Development Goal 5 [29]. Examples of policy documents developed by the Ministry of Health include the National Health Policy (2007), the Reproductive and Child Health Strategy (2005 – 2010), the National Road Map Strategic Plan to Accelerate Reduction of Maternal and New-born Mortality (2006 – 2010) and the currently released strategic plan called One Plan which stipulates strategies to reduce maternal, new-born and childhood deaths in Tanzania [27]. All these documents point to delivery of emergency obstetric care (EmOC) as an important component in reducing maternal mortality and morbidity (Table 1). However, effective implementation and provision of maternal health services such as EmOC depends on good health system governance.

**Table 1:** Activities focusing delivery of EmOC in district as stipulated by one plan document: 2008–2015 (MOH, 2007)

**Indicator for EMOC delivery**	**Activities**
Strengthen all dispensaries and health centers to provide Basic Emergency Obstetric Care (BEmOC)	• Deploy health workers (nurse-midwives, clinical officers and laboratory assistant)
	• Provide essential equipments and supplies for BEmOC
	• Build/Improve Infrastructure for service delivery (delivery room and postnatal wards)
Strengthen the capacity of district hospital and upgrade by 50% health centers to provide Comprehensive Emergency Obstetric Care (CEmOC)	• Deploy skilled health workers (Nurse midwives, Medical officers, Assistant Medical Officers, Anesthetists, Laboratory technicians)
	• Provide essential equipment and supplies for EmOC
	• Build/Improve infrastructure for service delivery (Operating theatres, labor wards, blood storage facilities, incinerators)
Strengthen health workers competencies	Develop and conduct tailor made training for nurse midwives and clinical officers at dispensaries to provide EmOC and for nurse midwives and assistant medical officers at health centers and district hospital to provide CEmOC

Health system governance, which entails the capacity to formulate policies, manage resources and provide service [30] is currently considered to be the determining factor for performance of the health system. It has been determined that poor health service delivery is associated with weak health system governance, lack of implementable plans or a shortage of human resources [31].

With decentralization, the CHMT shares governance responsibilities for the health system at the district level with different actors [26], and their working interaction influences the outcome of the policy implementation [32]. The effect of shared governance of the district health system as a result of decentralization is not well known. Furthermore, very little is known about how key health policy implementers at the district level experience implementation of EmOC.

Effective implementation of these activities depends on how CHMTs work with other partners to plan, organize and coordinate interventions at the district level. With the need to meet international goals of reducing maternal mortality by 75% in 2015 [33] and the national target of reducing maternal mortality from the current figure of 454 to 164, this study aims to explore the experiences and perceptions of CHMT in working with multiple partners while illuminating some governance aspects that affect implementation of EmOC at the district level.

## Methods

### Study setting

This study was done in Kongwa district in the Dodoma region of Tanzania. Kongwa was selected because it is a typical rural district. The district has 14 Wards, 67 villages and 286 hamlets. The total population of Kongwa District is 295,195 people according to the 2002 population census projection. Of these, 132,838 are males and 162,357 are females. The annual growth rate is 2.4% [34]. The health care system in Kongwa consists mainly of government owned facilities, with very few health facilities owned by the private sector and NGOs. The government owned facilities include one district hospital located at Kongwa capital, four rural health centres and 32 dispensaries. All these facilities provide antenatal care (ANC), deliveries and postpartum care. They also provide basic emergency obstetric care (BEmOC). Comprehensive emergency obstetric care (CEmOC) is provided in one rural health centre and at the district hospital. Pregnant women identified with complications or who are labelled as “at high risk” (due to being identified by health workers during antenatal care at dispensaries and rural health centres) are referred to the district hospital and can stay at the maternal waiting home (Chigonela) located at the district hospital while waiting for delivery. The CHMT is responsible for the daily activities of the district health system. The CHMT is answerable to the Council through the Council Health Service Board. It has a link with the MOHSW through the RHMT on technical and professional issues, and to the Zone Training Centres on capacity building issues.

The Kongwa CHMT consists of five core members, which are the District Medical Officer (DMO), District Nursing Officer (DNO), District Health Officer (DHO), District Health Secretary (DHS), and District Pharmacist (DP). The team can also have other co-opted members, such as the District Reproductive Health Coordinator (DRHCO). Co-opted members are invited by the team during discussions and/or implementation of specific activities [35]. The DRHCO is responsible for daily reproductive health activities including maternal health services taking place in the district. A review of planning documents and reports indicated improvement in some maternal health service indicators in the district between 2008 and 2011 (See Table 2).

**Table 2:** EmOC delivery indicator before 2008 and at the end of 2011

**EMOC delivery indicator**	**Period in relation to implementation**	
	**Before 2008**	**At the end of 2011**
Government Facility that conduct delivery with basic EmOC (Dispensaries, health centers and hospital)	30	37
Government Facility that conduct delivery with Comprehensive EMOC (Health centers and Hospital)	1 hospital	2 (1 hospital and 1 health center)
Maternal waiting home	0	1 at the district hospital
Number of skilled health workers in all facilities (Doctors, Assistant medical doctors, clinical officers, nurse midwives, laboratory technicians, anesthetist)	109	180
Number of ambulance for referral transport	2	5
Number of facilities with mobile phone for communication	0	37

### Data collection techniques

Different methods were used to collect data for this study. These included interviews and focus group discussions with members of the CHMT, interviews with key partners who work with the CHMT, observation, facility survey and documentary reviews (Table 3).

**Table 3:** Data collection methods

**S/N**	**Source of data**	**Quantity of data**
1	Individual interviews (N = 13)	Five Members of CHMT
		Four Health facility in charges
		One member of CHSB
		One member of RHMT
		One local government officials
		One member of NGOs
2	Documents	Health sector strategic plans
		Council health planning documents
		Council health reports
		Local government documents
		Guidelines for establishment of facility health committees CHMT and CHSB
3	Observation	One regional maternal audit meeting
		Regional meeting reviewing CCHP preparation
4	Focus group discussion	One focus group with 10 members of CHMT

Thirteen interviews and one focus group discussion were conducted with key informants concerned with implementation of EmOC. The interviews and focus group discussion were guided by an interview guide that included questions aimed at exploring the experience of respondents in implementing EmOC, their experience of working with multiple partners and how they perceived the role of each partner in EmOC implementation. The interviews were carried out in such a way that the interviewers asked open-ended questions, allowing respondents to narrate their experiences without any interruption. However, the interviewer, who is the first author, maintained the focus of the discussion through probing questions and asking for clarification. All the interviews and focus group discussion took place at the respondents’ work place and lasted about an hour, and were audio-taped with the help of a moderator. Notes and memos were written up both during and immediately after the interviews were conducted and recorded.

The working process and relationship between the CHMT and other partners in the district health system was observed in two meetings conducted during the period of data collection. One meeting involved the RHMT, the CHMT, the heads of health facilities and NGOs discussing challenges faced in preparing health plans. The other meeting involved the RHMT, the CHMT and NGOs discussing strategies on how to reduce maternal mortality.

Selected documents were reviewed based on their relevance in contributing to the overall themes being explored. The documents reviewed were the Tanzania Health Policy, Tanzania Health Sector Strategic Plan III (2008–2015), the Strategic Plan for Acceleration of Reduction of Maternal, Neonatal and Child Deaths (2008–2015), Council Health Reports (2007–2011), planning documents (2008–2011), Local Government documents and the Guidelines for establishment of CHSB and Committees. Review of these documents aimed at understanding the working structure, roles and links of partners and directions from the central authority on implementing policies related to EmOC at the lower level of the health system, the district.

### Data analysis

Audio taped interviews were first transcribed by the first author and translated from Kiswahili to English. All the material was analysed using qualitative content analysis, following Graneheim and Lundman [36]. The transcripts, field notes, observations, reports and reviewed documents were first analysed manually by reading and re-reading to become familiarized with the data. Transcripts were analysed for identification of text (meaning units) related to the processes, experiences and perceptions of partners on working together in implementing EmOC. The meaning units were condensed and codes were then extracted. Similar codes were sorted to form categories reflecting the manifest content of the text and similar categories were organized into themes reflecting the latent context of the text. Data from field notes, documentary reviews, observations and health facility surveys were used as supportive information in clarifying concepts that emerged during the content analysis process.

### Trustworthiness

Trustworthiness in the study is achieved when the findings are worth believing [37]. Being in the field for more than one month and being involved in data collection activities helped the first author capture the reality of those being studied. Collected data were also shared among co-authors, who gave critical comments and suggestions. Furthermore, the data collected from interviews and focus group discussions were triangulated with those from field notes, documentary reviews and observation during the analysis process to strengthen the validity and credibility of the findings.

### Ethical considerations

The study was ethically approved by the Senate Research and Publication Committee of Muhimbili University of Health and Allied Sciences. Permission to conduct the study was given by the Dodoma Regional Administrative Secretary (RAS). Informed consent was obtained after the researchers explained the purpose of the study. Participants were informed of their right to refuse participation in the study and were assured of the confidentiality of the collected information.

## Results

During analysis of the data, nine categories emerged that were cross-cut by two broader themes. The themes and related categories are presented in Table 4 below.

**Table 4:** Categories and themes emerging from the study

**CHMT experience on**	**Categories describing manifest content**	**Themes describing latent content**
EmOC implementation	• Making progress towards better services	A process accompanied by achievements and challenges
	• CHMT taking a lead and work with team spirit	
	• Increased demand for services	
	• Resource scarcity in term of skilled health workers, funds and time	
	Working with competing needs	
Working together with partners	• Acknowledging importance of partners	Partnership is necessary to make things happen
	• Partners play different roles	
	• A need for clear working arrangements	
	• A desire for community participation	

### A process accompanied by achievements and challenges

#### Making progress toward better services

Generally the CHMT viewed EmOC implementation as a step-by-step process that involves planning and organizing activities to be implemented while working with emerging challenges that act as obstacles towards the intended achievements. Respondents reported on several activities implemented, focusing on EmOC, and were satisfied they were making progress towards better services. They were also satisfied with the current situation in maternal health services. They were optimistic that the interventions made would help reduce maternal deaths. In expressing their feeling on the progress the team has made, one of the CHMT members said:

*Now the situation is very good! It is satisfactory and we are satisfied! It is good. We lost many mothers in previous years because of poor services. By then our services were not yet improved. At least now the situation is very good, there is no possibility of mothers losing their lives, or else it is up to them if they don’t follow instruction that we are giving them. Services are good now….* (CHMT member 1)

#### CHMT on taking a lead and work with team spirit

The CHMT described several steps they used to implement activities, focusing on EmOC while taking a leadership role in the whole process. As a leader, the team had to develop a clear health plan and prepare a budget for the activities to be implemented. The health plan prepared, known as the comprehensive council plan, has to include all health activities to be implemented, including EmOC activities. Preparation of the plan has to consider national health priorities and health needs from communities that are reflected in the health plans developed by each facility in the district. Apart from developing and sharing the plan, the team had to use several mechanisms to ensure they obtained all necessary inputs needed to achieve their goals. One mechanism the team used was that of lobbying to get nurses to work in their facilities by visiting nursing colleges, talking to student nurses directly and asking them to come and work in the district. An effective lobbying process was also used by the team to get ambulances for the transport of referred patients. The team also mentioned convincing and negotiating with influential people whenever they had a new idea, in order to win their consensus. One respondent narrated how the team managed to convince the district director and the council to set a priority of building a new health centre to provide Comprehensive Emergency Obstetric Care in a place where others thought it was not important to do so.

*You know as a leader sometimes you can involve and share with those in authority formally and informally. You can have a certain idea and you find the director in the office sometime before starting a meeting, you tell the director “look we have one, two, three things here… what do you think?” …, from there you go to those whom we call decision makers, the board chairperson and tell him that we have one, two, three ideas. For example, when we thought of building the K health centre, we met local leaders and explained to them using data on the importance of building K health centre as a priority; they became satisfied and started to advocate for it.* CHMT member 2)

#### Increased demand for services

The team reported an increased demand for services following community sensitization about institutional delivery. Respondents also reported on an influx of women from neighbouring districts after one of the rural health centres started conducting caesarean sections. While increased institutional delivery was considered as one of the achievements the team had attained, the imbalance between the increased demand for the service and decreased capacity to serve these women due to a shortage of health workers and funds posed a great dilemma for future EmOC delivery in the district. One respondent expressed this dilemma this way.

*We sensitized pregnant mothers to come and give birth in health facilities to minimize home delivery. The response is so high, and the way I see it, demand is becoming higher than our capacity when it comes to service providers. Sometimes one nurse stays the whole night with a mother at the dispensary and the same nurse is needed to provide care the following day to other mothers and other patients. So there is low capacity but still the pregnant mothers keep coming to the health facilities.* (CHMT member 3)

#### Resource scarcity in terms of skilled health workers and funds

A shortage of health workers was mentioned as one of the major challenges faced in implementing EmOC services in this district. Reviewing health plans and discussion with the team did not indicate any tangible plan on how the challenge of increased demand for institutional delivery is going to be dealt with. Furthermore, nothing was mentioned by the respondents as to how the existing health care providers were prepared to handle increased tasks so they do not compromise the quality of service provided. Also, none of the respondents from the team mentioned mechanisms to motivate health care providers who might be extremely exhausted as a result of increased workload.

Respondents indicated lack of sufficient funds as another challenge impairing EmOC implementation in the district. The team cancelled all trainings that were used to increase health providers’ competencies, especially to those who are newly recruited, due to budget constraints. Also as a result of budget constraints, the team failed to conduct maternal death review meetings, which were useful in tracing causes of maternal deaths occurring in the district and planning remedies accordingly. Furthermore, respondents described frustration over delays in the disbursement of funds from the central government, which put the team in the difficult situation of meeting running costs such as buying fuel for ambulances. This was described by respondents as one of the barriers towards implementing a no-charge policy in maternal health. Some respondents reported the difficulty they faced offering free maternal health services in the presence of budget constraint, and claimed that it was not realistic in actual implementation. They argued the government should disburse sufficient funds to fill the gap, as described below.

*Maternal health services should be free. But you know what…when we say they are free it means the government must contribute….must fill the gap. Because at the end of the day you find that you are struggling to offer services appropriately for free though the budget is very little…. When this happen it contributes to the provision of services with low quality, leading to decline in service delivery and the quality of service goes down* (RHMT member)

#### Working with competing needs

Working with competing needs from other health programs posed challenges to the team over effective EmOC implementation. Respondents expressed that, apart from EmOC, the team was also tasked to implement other health interventions such as Malaria and Tuberculosis programmes, which are sometimes better funded than maternal health programs. These created competition in terms of human resources, funds and time, further worsening the resource scarcity already existing in the district.

*We have many health interventions here in the district, such as for TB, Malaria and others. This causes a burden to us and to service providers. It is a lot of interventions and to manage them all is a real challenge. Other intervention’s donors invest a lot of money and seem to be very active, for example Malaria TB, and HIV, while others such as maternal health programs are less funded so sometime we have to support them from our own resources.* (CHMT 2)

### Partnership is necessary to make things happen

#### Acknowledging the importance of partners

CHMT respondents stressed the usefulness of different partners in EmOC implementation and perceived them as important players that together make a team be successful. In expressing this, one of the respondents claimed.

*I think it has been so helpful being together as a team; also working together with various partners has been helpful, because there are other areas in which we cannot play as a CHMT team. But after we involved several other stakeholders, things became easier. We have succeeded just by working together. Things would be different if we were working alone, yaah*! (CHMT member 5)

The CHMT perceived their limitation and considered working together with others as a mechanism to hasten effective implementation of EmOC, as commented on by one of the participants.

*I know strategies for improving maternal health services are there, but we need to join hands with others to make this happen*…. (CHMT member 6)

#### Partners play different roles

Partners played different roles during EmOC implementation. The regional health management team (RHMT) provided technical advice on how EmOC should best be implemented in the district. Heads of other sectors in the district supported the CHMT in better planning their implementation by commenting critically during preparation of the health plan. NGOs and development partners were mentioned to complement the resource gap during EmOC implementation through adding funds to meet running costs or supplying essential EmOC equipment to the newly built health facilities. Local politicians and religious leaders were reported to have sensitized the community on use of the health facilities. Of interest is the role of men and Traditional Birth Attendants (TBA) that was mentioned during interviews with respondents. As mentioned by respondents, men have been encouraged to escort their wives to health facilities during antenatal visits, and service providers used that opportunity to teach them about safe birth preparedness procedures. TBAs were mentioned to be useful agents in increasing institutional delivery, especially since they had been allowed to escort pregnant mothers to health facilities during delivery. CHMT respondents took male partners and TBAs as potential actors in facilitating the continuum of care even during the postpartum period. However, TBAs were not involved in the planning process for the health plan even at the community level. Furthermore, the health policy documents reviewed did not mention TBAs as important partners in maternal health.

#### A need for clear working arrangements

Given the number of partners the CHMT worked with, a concern was raised as to the need for working arrangements for effective partnership. Review of documents revealed unclear working structures, mixed roles and links and a flood of directions from the central authority that affect the working relationships of partners in implementing policy related to EmOC at the lower level of the health system, the district. Respondents recommended policy documents need to be clear, to stipulate each partner’s roles and to give clear working links among partners to guide partnership in service provision.

*Another thing is involvement of others, because if only the CHMT is involved the burden will be large but if all partners collaborate and if there would be guidelines that link with other actors/partners, it will help to put people in correct alignment so that we may also offer good services. It can be sustainable only if we have clear guidelines and these guidelines are given a hundred percent recognition, it would be much better. Each one would know his/her role directly.* (CHMT member 7)

#### A desire for community participation

In addition to this, the respondents indicated a desire to have effective community participation through active health facility governing committees. Respondents viewed these user representative committees as important partners in overseeing service delivery at the facility level. However, these committees were described to be weak and not performing as intended.

*These committees are responsible for overseeing resources at the centre. When drugs arrive at the health centre, they are supposed to call themselves and approve drugs received at the health facility. Even expenditures at the centre cannot be done without their approval. You will find that when the facility in charge needs them, they are not available or if they come, they don’t give any help. They are just there as if they are not responsible. However it will reach a point where these committees will consist of educated people with better understanding than those who are present now. But it is also a matter of the whole community in general; when the community is sensitized we will get good people who are very active in the committee*. (CHMT member 2)

## Discussion

This study found that a decentralized health system resulted in that a number of partners were involved in governing implementation of emergency obstetric care. Effective implementation of EmOC depends on how the central government, the local government, development partners, NGOs and the community work with the CHMT and whether this working interaction is guided by a framework that holds each partner accountable and responsible.

### The leadership and stewardship role of the district health management team in maternal health policy implementation

Despite the involvement of many players in policy implementation, the district health management team holds an important lead role. The CHMT had to use leadership skills such as sharing, negotiations, lobbying and coordination to align different actors found in the district in implementing EmOC. Acting as a middle man between the central government and different actors in the district, the team translated national policies into a plan with consideration for local demands, shared and negotiated the plan with local leaders, lobbied for inputs and coordinated all important actors for optimal implementation. These findings demonstrate that effective implementation of health related policies is not a smooth process and requires local health managers to exercise leadership skills to effectively work at the complex district health system governance structure. The WHO in its framework for strengthening health system stipulated leadership/governance (stewardship) as one of crucial building block in improving performance of the health system [38]. Our results have also noted the central role of the Ministry of Heath of formulating a strategic vision that CHMT used to guide development of the health plan which is to be implemented at district level. The WHO report 2000 pointed out three main tasks of the government as a steward which include i) formulation of health policy, ii) setting regulations and iii) collection and use of necessary information to inform decision making [39]. The stewardship is considered to be the steering role of the health system [40] and an important component for better performance [40-43]. However, with decentralization, the stewardship role of Ministry of Health is partly delegated to local actors, in our case, the CHMT. While there is concern of mismatch between responsibilities given and powers provided to the CHMT to execute their roles [41], our study further demonstrates the role of local stewards in improving local health system performance. Findings from this study further suggest that for the better function of the health system at local level, the stewardship responsibilities of the CHMT should match the powers bestowed to them.

Empirical studies done elsewhere also described the link between district health managers and health system performance. A study done in Pakistan revealed that district health managers contributed to reducing maternal deaths through successfully coordinating implementation of maternal health policy [44]. Studies conducted in Zimbabwe and Kenya indicated the link between underperformance of the district health system and ineffective district health management teams [45,46]. This imply that the district health managers, who act as local stewards of health system are crucial actors to effectively overseeing and guiding implementation of health policy designed at the central level and how they execute this role can shape performance of health system at the local level.

### Accountability of the central government to local implementation of maternal health

Emerging challenges during policy implementation hampered the efforts of the district health management team and could contribute to delays in achieving the policy. A shortage of skilled health workers, financial constraints and delayed disbursement of funds from the central government were indicated as impediments to effective implementation. Competing needs from other health interventions and conflicting interests of NGOs and donors, together with the bureaucratic decision making process in a decentralized health system, further complicate the whole process. Other studies have reported the same challenges related to effective implementation of health related policies [47-50]. Idd and colleagues indicated that implementation of pro poor exemption policy in Tanzania, which intended to make health services more accessible to poor people and vulnerable groups, was hampered by contextual factors at the district level and specifically the practices of district health managers [48].

Therefore, the government should ensure that, the district health managers are well trained on managerial skills necessary to carry out emerging roles needed to effectively implement policy change. The government should scrutinize the training that is currently offered to CHMT to see if it is effective in meeting the demand of developing local health leaders’ managerial competencies and skills. With decentralization, the local health leaders have been required to execute managerial skills such as financial and administrative skills, although the current training programmes do not offer them an opportunity to acquire such skills. Lack of managerial skills of local health leaders together with limited financial resources, lack of skilled health workers and political interference has been linked to unsuccessful implementation of health programs in developing countries [5,51-54].

Frameworks guiding implementation of health policy, like those suggested by Hercot and McPake and their colleagues could be considered to ensure effective policy implementation [55,56]. These frameworks describe a sequential step by step process which should be followed for successfully policy implementation. Both proposed frameworks emphasize the importance of conducting situation analysis to identify contextual factors that would hinder effective implementation of policy. Learning from the experiences of other countries implementing similar policies has been also recommended as a useful step towards achieving better policy outcomes [55,56].

Some countries have demonstrated excellent achievements in reducing maternal mortality and improving maternal health in general amid the resource constraints experienced [9]. Dramatic reductions in maternal mortality in China between 1998 and 2005, from 125 to 68 maternal deaths per 100,000 live births, was reported to be contributed by the central government’s visible commitment to supplementing local officials’ inputs in resource allocation to subsidize institutional deliveries, and meeting cost deficits for transport and referral [57]

With this in mind, our findings suggest the need for support from the central government in dealing with challenges encountered by district health managers once the newly developed policy is implemented at this level. As shown by Nimpagaritse and Bertone [50], the central government sometimes overlook challenges faced by key policy implementers at the district level, giving an example of how abolition of user fees in Burundi resulted in financial crisis and increase of administrative workload leading to reduced quality of services. Findings from our study urges thorough involvement of key implementers from the district level during development of health policy and the government’s full support to back effective implementation of the developed policy.

Establishing accountability measures for delays in fund disbursement and increasing the budget by the central government to meet running costs could hasten the achievement of planned activities. Furthermore, implementing a mechanism for motivating existing overloaded health workers and increasing the recruitment of new health workers with attractive packages are some governance measures that could be taken by the central government to ensure effective implementation of EmOC at the district level. However, motivation needs to be coupled with an enabling organization structure and clear human resource policy. A lack of clear accountability mechanisms at the district health system level and minimal inclusion of health workers affairs in developed health policies are mentioned elsewhere to be major central governance related obstacles that hamper policy implementation at the local level [58-60].

### Partnership in maternal health implementation at the district health system

It was found in this study that the CHMT worked closely, not only with the central government, but also with other partners found in the district, including local government officials, development partners and NGOs. Given the complex nature of maternal health problems, this working relationship is needed for efficient EmOC implementation.

Local political leaders elected by the community have an influential role in policy implementation. With decentralization, local politicians became major decision makers and are responsible for the allocation of funds at the district level. Understanding of the policy intentions by these actors would facilitate smooth implementation and better policy outcomes. Findings in this study indicated the involvement of local political leaders in EmOC implementation through approving budgets for EmOC activities, sensitizing the community to utilize the facility for delivery and mobilizing funds for building houses for newly recruited health workers. The sharing of developed health plans by the CHMT during council meeting gave these local political leaders the chance to understand what activities were suggested for implementation and share these responsibilities together with the team. It was learnt from this study that the CHMT, which is basically made up of technical experts, has to learn how to translate dispatched policy from the central government and make it understandable to local implementers. Many of these policies are huge documents, written in technical language and difficult to comprehend. Furthermore, the CHMT has to learn how to communicate with people outside their cadres and develop the ability to market these policies to gain the political will of decision makers in the district.

NGOs and development partners supplemented resource gaps by supplying facilities with equipment needed for emergency obstetric care. However, most of these NGOs and donors worked through short-term projects. Managers of the health system should plan ahead of time how they would sustain EmOC service delivery without support from development partners. NGOs and developing partners found in rural areas also direct funds to other vertical programs such as Malaria and HIV, leaving maternal health programs under funded. A study done in Uganda showed that donor-driven policies and donor-funded programmes significantly affect the way in which health care is provided, leading to a distortion of priorities and modes of service delivery [61]. It is then the role of the CHMT to ensure that donor pressure is not affecting their overall focus while creating a harmonized environment and good working relationships, something challenging in most developing countries.

### Community participation and local accountability in maternal health implementation

Implemented EmOC services should be of good quality, accessible, available and acceptable by users. This is crucial, especially when long term impact is intended, in this case reductions in maternal mortality and morbidity. To achieve this, technical strategies laid down by CHMT should be linked with community participation and local accountability. The involvement of TBAs, male partners and facility governing committees, it was found in this study, signalized the importance of mutual responsibility and accountability between the health system, government and communities in improving maternal health.

Several studies have reported on the need to integrate traditional medicine into the formalized health system [62-64]. In the same way, the involvement of male partners and their integration in maternal health would give them greater responsibility for the women’s health and strengthen the continuum of care as advocated by a new approach of partnerships for maternal and newborn health [65,66]. However, the sustainability of this working relationship is jeopardized by a lack of policy that recognizes the emerging role of TBA and male partners in EmOC and the absence of a framework that integrates them in the formal health system.

Health facility governing committees (HFGC) are needed to improve quality services delivery, either by overseeing facility resources or holding accountable health service providers. However, many health facility governing committee were reported to be weak, raising CHMT concern over their governing functions. Weak governance processes or lack of governance structures and systems at the community level minimize women’s engagement in health related decision-making [67]. While during this study the CHMT perceived HFGC as their helping hand in governing health facilities, these committees were established to provide a forum for community opinions and needs to be incorporated in health planning [68]. An effort should be made to alleviate patronage view of CHMT over these committees, a problem which is common under decentralization [59]. Instead, implementation of the EmOC agenda should go hand in hand with empowering communities and their representatives in HFGC, if increased community participation and local accountability to service provision are really desired [10].

### Study limitation

We recognize the limitation of not exploring the experiences of service providers and service users. Their voices are important and might have added different perspectives. Further research should include theirs and other stakeholders’ views and experiences. Qualitative research aims for analytical generalization. The insights gained from this study might provide an insight of the EmOC implementation in other districts given the similarities of how the district health system in Tanzania is organized. We have tried to describe the context in details to enable the readers to better judge the transferability of our findings to similar settings.

## Conclusion

This study indicated that implementation of EmOC policy is a process accompanied by challenges that need multiple partners to address them. It advocates working together in partnerships to govern implementation of EmOC. However, to have effective partnerships, the roles and responsibilities of each actor should be clearly stipulated in a clear working framework within the district health system.

CHMTs are important actors in policy implementation at the district level. Being the leader of the district health system, they are responsible for translating policy received from the MoH into a plan, mobilizing resources, aligning different partners, and organizing and coordinating various activities during implementation of these policies. Empowering them to improve their leadership skills, such as negotiation and communications skills, will improve their capacity to create better working networks with the other partners.

Apart from formulating policy and guidelines, the central government should fulfil its obligations at the district level. The government should provide clear governance related interventions to deal with persistent health system challenges that hinder effective implementation of policy at the district level. These interventions include a) Establishing accountability measures for delays in fund disbursement. b) Implementing mechanisms for motivating overloaded health workers. c) Training and recruiting new skilled health workers and creating an attractive working environment, especially in rural areas. d). Budget allocation for the health facilities should be increased in line with the increased service demand from the community.

For efficient partnership in policy implementation and service delivery, a working framework needs to be developed with clear roles and responsibilities for each partner. Furthermore, a mechanism on how to integrate TBAs and male partners in improving maternal health needs to be formulated.

Community participation in service delivery through health facility governing committees would improve governance of the health services by the users. The CHMT should, as part of their agenda during EmOC implementation, create an environment where the community and its representatives are empowered to voice their concerns, hold service providers accountable and oversee health service provision for its quality.

## Competing interests

The authors declare that they have no competing interests.

## Authors’ contributions

DAM conceived the study, participated in its design, carried out interviews and analysis, and drafted the manuscript. AK participated in the design, was responsible for overall coordination and helped to draft the manuscript. IG and AKH participated in the design, analysis and helped to draft the manuscript. All authors read and approved the final manuscript.

## Pre-publication history

The pre-publication history for this paper can be accessed here:

http://www.biomedcentral.com/1472-6963/14/333/prepub
